# Lizards and tortoises show evidence of low inhibitory control

**DOI:** 10.1038/s41598-025-08373-9

**Published:** 2025-07-02

**Authors:** Maria Santacà, Anna Wilkinson, Gionata Stancher, Valeria Anna Sovrano, Angelo Bisazza

**Affiliations:** 1 Department of Behavioural and Cognitive Biology, University of Vienna, Djerassiplatz 1, 1030, Vienna, Austria; 2 Department of Life Sciences, University of Lincoln, Lincoln, UK; 3 Rovereto Civic Museum Foundation, Rovereto, Italy; 4 Center for Mind/Brain Sciences (CIMeC), University of Trento, Rovereto, Italy; 5 Department of Psychology and Cognitive Science, University of Trento, Rovereto, Italy; 6 Department of General Psychology, University of Padova, Padova, Italy; 7 Padua Neuroscience Center, University of Padova, Padova, Italy

**Keywords:** Cylinder test, Reptile cognition, Motor Inhibition, Comparative cognition, Detour-reaching behavior, Animal behaviour, Cognitive neuroscience

## Abstract

Inhibitory control, the ability to suppress an automatic response in favour of a contextually appropriate alternative, is crucial for adaptive behaviour across animal species. While extensively studied in mammals and birds, research on reptiles remains limited, with comparisons hindered by methodological inconsistencies. Here, we assessed inhibitory control in two reptile species, Hermann’s tortoise (*Testudo hermanni*) and the bearded dragon (*Pogona vitticeps*), using the transparent cylinder test—a method widely employed with mammals, birds, and fish. This test evaluates the ability to inhibit reaching directly for visible food through a transparent barrier. Both species exhibited lower inhibitory control than most amniotes, supporting the prevalent hypothesis linking inhibitory capacity to brain size. However, exceptions observed in various species suggest ecological and non-cognitive factors also shape these abilities. Notably, bearded dragons outperformed tortoises, despite their smaller size. In tortoises, females surpassed males, highlighting sex-based inhibitory differences in non-avian reptiles. Additionally, a positive correlation between lateralization and inhibitory control was observed in both species, providing the first evidence of such a link in reptiles. These findings emphasize the role of lateralization in reptilian cognition and suggest that inhibitory control across vertebrates is influenced by diverse factors, including brain size, ecology, and sex.

**Supplementary Information:**

The online version contains supplementary material available at 10.1038/s41598-025-08373-9.

## Introduction

Inhibitory control refers to the capacity to suppress a prepotent or automatic response in favour of a more context-appropriate alternative, playing a crucial role in adaptive behaviours. For instance, ambush predators delay their attack until prey is optimally positioned^1^, while prey species restrain feeding in the presence of threats^2^. In hierarchically structured primate societies, subordinates inhibit feeding or sexual responses in the presence of higher-ranking individuals^3^. These examples illustrate different instances of inhibitory control, which encompasses various processes such as response inhibition, delayed gratification, and behavioral flexibility—capacities that are often assessed using standardized experimental paradigms.

Inhibitory capacities have been studied in a wide variety of organisms. While some studies have been conducted in natural settings^4,5^, most research on inhibitory control in animals has been performed in the laboratory. The transparent cylinder test is a benchmark method for studying inhibition. It can be easily administered to diverse species, allowing direct comparisons even between phylogenetically distant animal species. In this test, an individual is first trained to retrieve a food reward from an opaque cylinder that is open at both ends. Once the subject reliably performs this task, the opaque cylinder is replaced with a transparent one. The test evaluates the individual’s ability to inhibit the motor impulse to reach directly for the reward through the side of the cylinder, which acts as a transparent barrier, and instead navigate to the open ends to obtain the reward. The opaque phase is intended to allow animals to acquire the correct motor response, thereby minimizing the contribution of learning processes during the test phase. It also ensures that subjects are motivated and comfortable with the setup, helping to distinguish genuine inhibitory failures from responses influenced by neophobia or low engagement. Although widely used, the transparent cylinder test has been criticized for its sensitivity to small methodological changes and non-cognitive factors that can affect performance across individuals and species (reviewed in^6^). For instance, prior experience with transparent or semi-transparent barriers might facilitate the recognition of the cylinder as an obstacle^7,8^, and it is therefore important to control for this factor when comparing performance across studies and species. Sensory differences can also influence how animals perceive the task, a factor that becomes particularly relevant when comparing phylogenetically distant species. For example, species that rely heavily on olfactory cues may perform differently than those that depend primarily on visual input, given that the transparent barrier is visually permeable but olfactorily opaque^7^. Such factors, though often overlooked, can introduce significant variability that is not necessarily indicative of differences in inhibitory control per se. Acknowledging these influences is thus crucial for interpreting interspecific comparisons in performance.

Historically, research has focused on mammals and birds, revealing considerable interspecific variation in inhibitory capacity, with certain groups, such as primates and corvids, outperforming other groups^9,10^. A large-scale, multi-lab study on 36 mammal and bird species found that the best predictor of performance was absolute brain volume^9^. Inhibitory capacity also correlated with general intelligence (or g-factor), and complex cognitive abilities such as innovation, tool use, and social learning. They suggested that the reason inhibitory control correlates with brain mass is that larger brains have greater computational capacity. However, brain mass alone is only a loose predictor of a species’ information processing capacity, as neuronal density varies significantly across taxa^11^. Herculano-Houzel^12^ re-analysed the data sets from MacLean and collaborators^9^, along with a subsequent study on three corvid species^10^, identifying the total number of cortical or pallial neurons as a stronger predictor of cylinder test performance. Additionally, ecological factors, such as diet, may further influence inhibitory capacities^9^. Species with broader diets tend to exhibit more advanced cognitive functions, as they face complex decision-making in varied foraging contexts. This may partially explain the superior performance observed in omnivorous species like corvids and terrestrial primates.

More recently, inhibitory control has also been investigated in teleost fish^13–15^. Interestingly, although teleosts have much smaller brains than birds and mammals, their performance in inhibition tasks is often comparable to that of endotherms. This apparent discrepancy suggests that the brain size–inhibitory control relationship may not apply uniformly across vertebrates. Some authors^16–20^ have proposed that the cognitive abilities of teleosts reflect unique neurobiological adaptations resulting from their long, independent evolutionary trajectory following their divergence from the lineage that led to terrestrial vertebrates.

However, because comparisons have so far been limited to teleosts and endotherms—two evolutionarily distant groups that differ markedly in brain organization and sensory ecology—this evidence alone is insufficient to resolve the question. To address this issue, data from other ectothermic vertebrates—particularly reptiles and amphibians—are critically needed.

No studies exist on cartilaginous fish, lobe-finned fishes or amphibians. Three recent studies have examined inhibitory control in reptiles^21–23^, offering valuable insights and highlighting interspecies differences. However, due to substantial methodological variation—particularly in test design and stimulus transparency—these findings are difficult to compare directly with the extensive body of work on other taxa. The first study focused on a free-living lizard (*Anolis sagrei*), using a modified version of the cylinder test that lacked an initial training phase with an opaque cylinder and employed a transparent apparatus with only a central clear section^23^. The second and third studies assessed five skink species, reporting species differences in motor inhibition, with blue-tongued skinks making fewer errors than other species^21,22^. However, these studies used a semi-transparent mesh cylinder rather than a fully transparent one and transparency levels are known to significantly affect performance in such tasks^24–26^.

Cerebral lateralization—the specialization of the left and right hemispheres for different cognitive functions—is widespread across all vertebrate classes^27^. In fish and endotherms, lateralized individuals have been shown to outperform non-lateralized ones in various cognitive domains^28,29^. Two recent studies investigated whether there is a relationship between cerebral lateralization and performance in inhibitory control tasks in waxbills and zebrafish^30,31^. In both cases, individual differences in lateralization were significantly associated with the degree of inhibitory control, raising the possibility that this effect may also play an important role in other species.

To start to fill the gap in the comparative literature, the present study investigates inhibitory control in reptiles using the exact same procedure of the transparent cylinder test that has been previously applied to mammals, birds, and fish, so to ensure methodological consistency and allow direct comparison across taxa. To this end, we selected two phylogenetically distant reptile species: a tortoise (*Testudo hermanni*) and a lizard (*Pogona vitticeps*). Both species are diurnal active foragers and have been the subject of several ecological, behavioural, and cognitive studies, offering a contextual basis for interpreting potential differences in task performance.

Previous research on bearded dragons showed a consistent eye preference when observing stimuli, suggesting hemispheric specialization in visual processing^32,33^. Similarly, in Hermann’s tortoises, lateralized behaviour has been observed in righting reflexes, with a preference for turning in one direction to regain an upright position^34^, as well as in interactions with mirrors^35^. These studies underscore the relevance of lateralization in diverse behavioural contexts, raising the possibility that in our two species it might also influence inhibitory control performance. In both species, we measured individual lateralization to assess whether this phenomenon also affects inhibitory performance. For tortoises, which had a larger sample size, we also examined sex differences.

## Materials and methods

### Ethical note

This study adhered strictly to ethical standards for animal research, ensuring that both tortoises (*Testudo hermanni*) and bearded dragons (*Pogona vitticeps*) experienced minimal stress throughout housing, testing, and post-experiment care. Animal husbandry and experimental procedures complied with European Legislation for the Protection of Animals (Directive 2010/63/EU) and the ARRIVE guidelines. The study with tortoises was carried out at the natural estate of “SperimentArea” (Civic Museum Foundation of Rovereto, Trento, Italy) and was done in accordance with the Italian and European Community laws on protected wild species (Art. 8/bis 150/92 all. A Reg. (CE) 338/97). The experimental protocol was authorized by the internal Ethics Committee of the Civic Museum Foundation of Rovereto (Prot. A N. 0000238 – dd 21/06/23). The study with bearded dragons was carried out at the University of Lincoln. Applicable national guidelines for the care and use of animals were followed. This research was approved by the ethics committee of the School of Life Sciences, University of Lincoln (protocol N. CoSREC364).

Animals were housed in enriched environments suited to their species-specific needs before, during and after the experiment (see below for details). For tortoises, testing was scheduled during the animals’ peak activity season, further reducing stress associated with temperature or seasonal inactivity. The testing protocol, the transparent cylinder task, relies on spontaneous observation of behaviour, with all our subjects participating voluntarily and showing no signs of stress. In each trial, animals were provided with a preferred food reward and received additional feeding post-trial. The sample size was determined by the maximum number of animals available for each species, given the practical limitations related to their slow growth and long lifespan. At the conclusion of the experiments, all animals were returned to their original enclosures, ensuring their well-being and continuity of care.

### Subjects

Experiments were conducted on a sample of 24 adult tortoises (*Testudo hermanni*, 12 males and 12 females) and 8 adult bearded dragons (*Pogona vitticeps*, 6 females and 2 males). Animals included in this study had prior experience with cognitive testing; however, none had been previously exposed to tasks involving transparent cylinders or detour obstacles. Tortoises were maintained in groups in an open-air enclosure at the SperimentArea, which is under the supervision of the Rovereto Civic Museum Foundation, located in Italy. The enclosure was partially bordered by wire mesh walls, composed of two different mesh sizes. During the study, the tortoises had unrestricted access to clean water and shelters. The experiments were run during the period spanning from June to July, corresponding to the months of higher activity of this species. We matched body size between the sexes as much as possible; indeed, we found no significant difference in their size considering the Straight Carapace Length (SCL): one-sample *t*-test, *t*_22_ = −0.086, *p* = 0.932. Bearded dragons were housed individually or in pairs in glass vivariums at the School of Life Sciences, University of Lincoln (UK). During the study, the bearded dragons had unrestricted access to clean water, shelter, UV light and heat lamps. All reptiles were not food deprived during the experiments; they received a favoured food reward during the experiment and were also fed after finishing the daily trials.

### Stimuli and apparatus

The stimuli consisted of small pieces of tomatoes and red cabbages for the tortoises and vegetable extract (kale, cucumber and mint) jellies for bearded dragons, highly preferred food for both species. Vegetables and jellies were freshly prepared and cut each day.

**Fig. 1:**
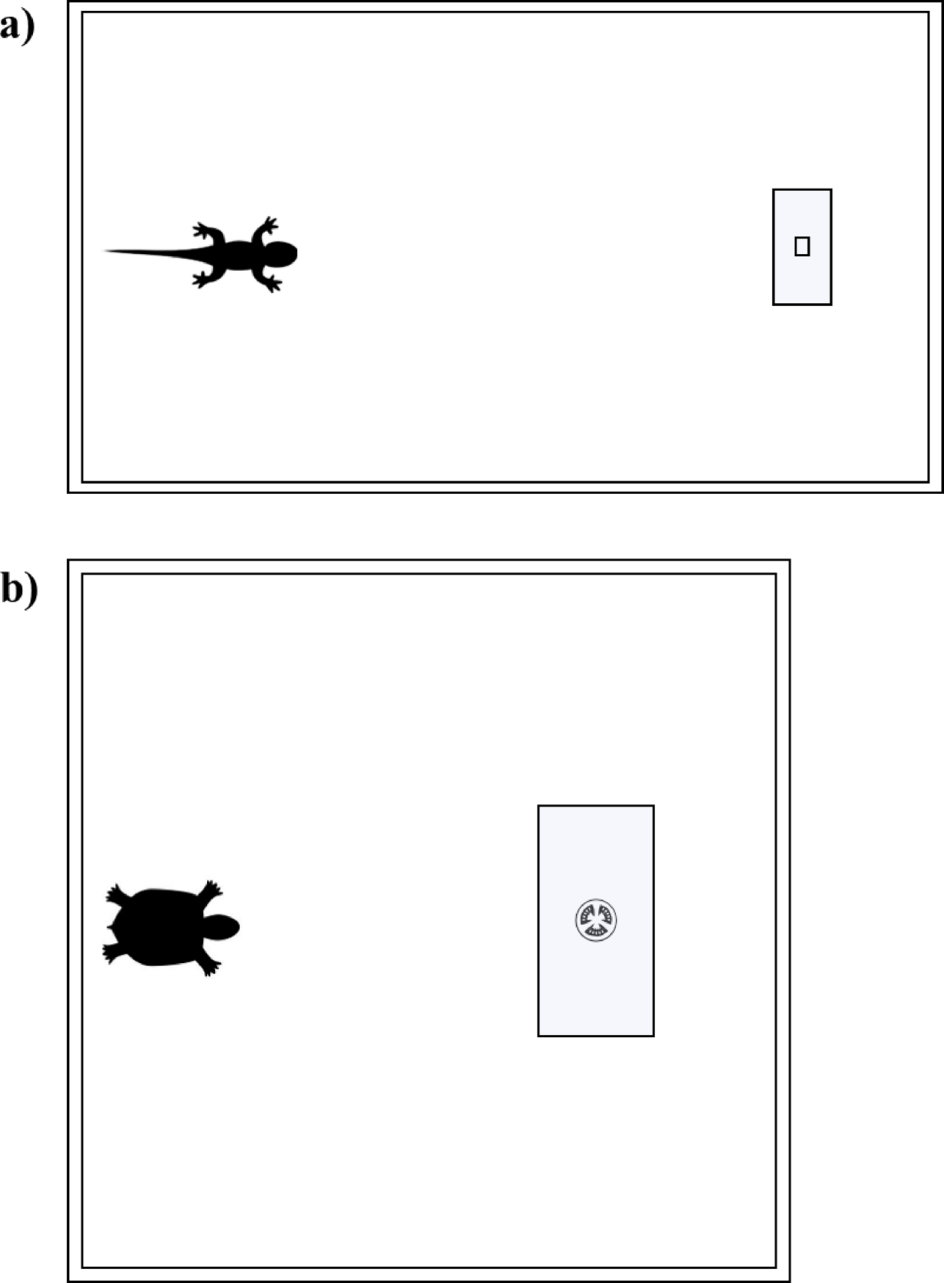
View from above of the apparatuses and transparent cylinders used for bearded dragons (**a**) and tortoises (**b**). The rectangles indicate the positions of the transparent cylinders and the placement of the food within them.

For bearded dragons, the apparatus consisted of a rectangular arena (110 × 61 cm; Fig. 1a) entirely covered with black plastic and was placed in an illuminated test room. For tortoises, the apparatus consisted of a wooden arena (90 × 90 cm; Fig. 1b) that was centrally lit from above (~ 150 cm) through a 400 W halogen lamp. The arena was placed in a dark room. The inner part of the arena was covered with wooden pellets. The experimenter was present in the room during the trials but remained outside the animals’ visual field to avoid influencing their choices. All tests were video recorded with a video camera placed above the apparatuses. For all reptiles, the cylinder test took place near an apparatus wall, opposite to where they were inserted into the test apparatus. We used two types of cylinders in the two different phases of the procedure: the training cylinder was opaque (green plastic), whereas the test cylinder was transparent (acetate). For bearded dragons, they were both 15 cm in length and 10 cm in diameter (Fig. 1a) whereas, for tortoises, they were both 30 cm in length and 20 cm in diameter (Fig. 1b). The size of the cylinders was selected to allow each animal to easily pass through with its entire body. All cylinders for both species were glued onto a plastic base (tortoises: 30 × 30 cm; bearded dragons: 15 × 15 cm).

### Procedure

#### Training phase

This phase lasted two days during which reptiles learnt to obtain food inside the opaque cylinder. The subjects performed five trials per day with a 30-minute interval between trials. Reptiles had 30 min to reach the food in the opaque cylinder; otherwise, the trial was considered null and was repeated. However, if the subject entered the cylinder, we waited 5 min before removing the cylinder to allow the subject to consume the food. All animals entered the opaque cylinder at least 4 out of 5 times on the final day of training, so no subjects were excluded as all demonstrated learning of the motor response.

#### Test phase

The test phase was similar to the training phase, but the opaque cylinder was replaced with the transparent one. In this phase, all reptiles received five trials per day for 4 consecutive days with a 30-minute interval between trials. Although the large-scale comparative study previously cited^9^ assessed all species using only 10 trials, subsequent research has shown that performance in later trials can also provide valuable insights into the cognitive processes under investigation^8,13,37^. For this reason, we extended the test phase to 20 trials to better capture intra-individual performance dynamics and to increase the power of intergroup comparisons across species and sexes. Based on the video recordings, we scored both the reptiles’ accuracy and the time required to enter the cylinder in each trial. For the accuracy, we scored a correct trial when the subject entered the cylinder directly via the lateral openings without touching the surface of the cylinder with its front limbs or head. Incidental contacts (e.g., belly, shell, or tail) were not recorded as errors. An entry was considered successful when the entire head of the animal was inside the cylinder. Instead, we scored an incorrect trial when the subject touched the transparent material before entering the cylinder. To score the time to solve the task, we measured the time from when the subject was inserted in the apparatus to when it entered the cylinder. In addition, we recorded the side (left or right) from which the animal entered the cylinder for each trial. One-third of the videos of each species were analysed by two different experimenters to assess interrater reliability. Trial-by-trial interobserver agreement was calculated by dividing the number of trials in which experimenters agreed by the total number of trials and then converting the result to a percentage; the mean agreement for total performance was 99%. Even the time to solve the task indicated high interrater reliability (Spearman rank correlation: ρ = 0.944, *p* < 0.001). Therefore, we conducted all the analyses with the database of the first experimenter.

### Data analysis

Analyses were performed in R version 4.3.2 (The R Foundation for Statistical Computing, Vienna, Austria, http://www.r-project.org) and the code used in this study is available in the supplementary material. We analysed the subjects’ accuracy in each trial (correct or incorrect) with generalized linear mixed-effects models for binomial response distributions (GLMMs, ‘glmer’ function of the ‘lme4’ R package). To assess whether accuracy increased day after day and trial after trial (within the same day), we fitted the models with the day (1–4) and trial number (1–5) as fixed effects. Moreover, we also fitted the time to reach the food (after log transformation) as a fixed effect whereas individual ID was fitted as a random effect. Only for tortoises (due to the balanced sample size), we also included sex as a fixed factor. Interactions among all fixed effects were tested.

To assess whether there was a population-level lateralization bias in the direction subjects entered the transparent cylinder, we calculated the proportion of entries from the right side for each individual. We then tested whether this proportion significantly deviated from chance level (50%) using one-sample two-tailed *t*-tests. For bearded dragons, a single test was conducted on the entire sample. For tortoises, separate tests were performed for males and females respectively. A Pearson correlation test was used to assess the correlation between the proportion of correct choices and a ‘lateralization’ index calculated as the proportion of entering the cylinder from the preferred side. A lateralization index of 1 means that a subject always entered the cylinder from the same side (always right or always left); instead, a lateralization index of 0.5 means that half of the time the subject entered from the right side and the other half from the left side suggesting no lateralization.

Sex differences in the time to enter the opaque cylinder (after log transformation) during the training phase in tortoises were analysed using a linear mixed-effects model (LMM, ‘lmer’ function of the ‘lme4’ R package) with sex as a fixed factor and individual ID as a random effect. To compare the training phase of the two species, we analysed the subjects’ time to enter the opaque cylinder (after log transformation) with a LMM. We fitted the model with the species and the day (1–4) as fixed effects and individual ID nested within species as a random effect. To compare the accuracy of the two species in the test phase, we fitted a GLMM for binomial response distributions with the species and the day (1–4) as fixed effects and individual ID nested within species as a random effect. The interaction between the two fixed effects was tested in both models.

## Results

### Bearded dragons

With the opaque cylinder, bearded dragons entered the cylinder in 98 ± 16% of trials (mean ± SD). The mean time required to enter the cylinder was 282 ± 362 s (mean ± SD).

With the transparent cylinder, bearded dragons performed 41.25 ± 28.26% correct trials (mean ± SD) in which they retrieved food without touching the transparent cylinder (correct trials). The mean time required to complete a trial was 145 ± 106 s (mean ± SD).

**Fig. 2:**
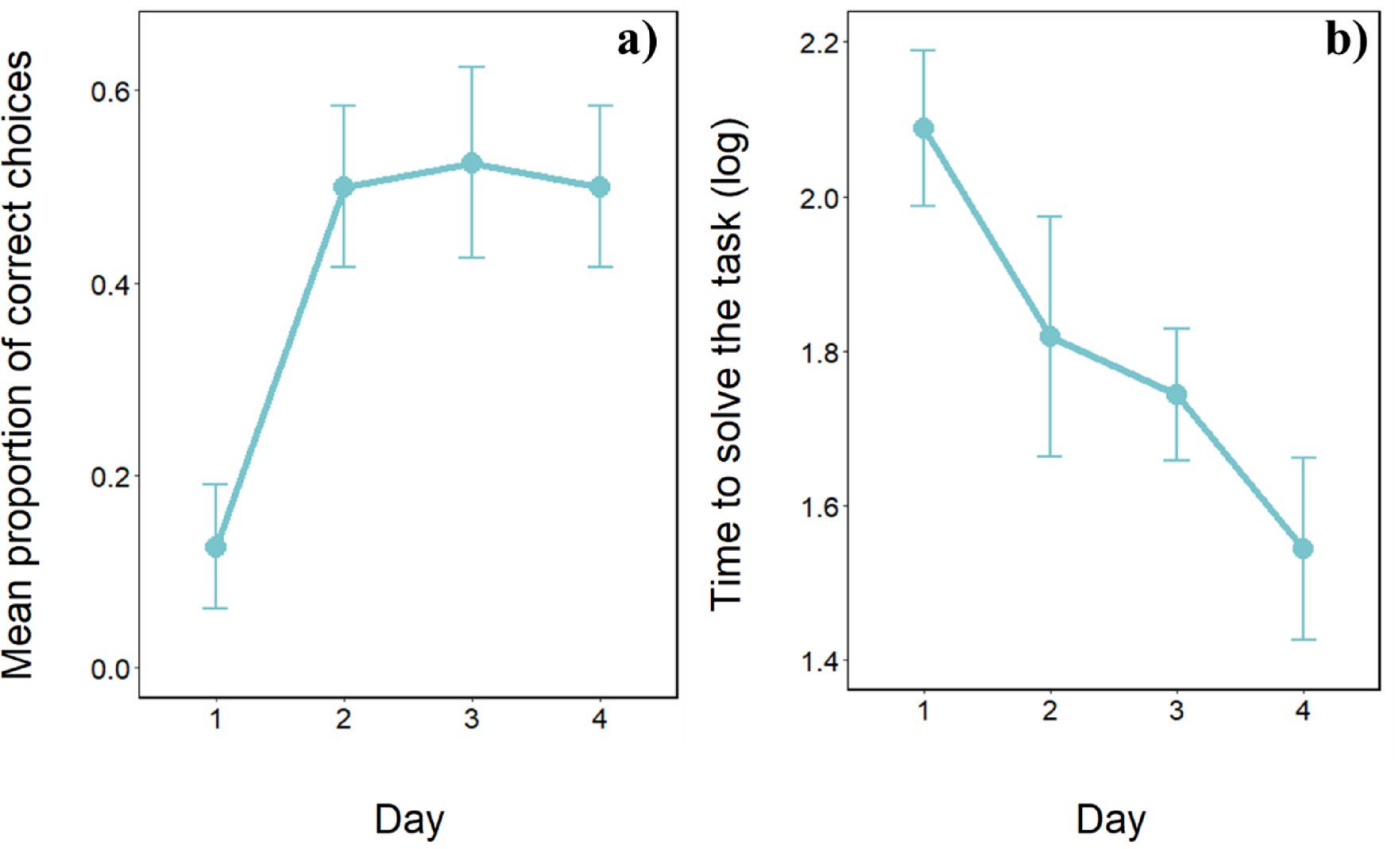
Performance of bearded dragons in the cylinder task. (**a**) Mean proportion of correct trials in which bearded dragons did not contact the cylinder (mean ± SE), and (**b**) time to solve the task (mean ± SE) over the four days of the test phase.

The GLMM revealed a significant effect of the time on subjects’ accuracy (χ^2^_1_ = 4.189, *p* < 0.05): the longer the time to reach the food, the lower the performance. Also, both day and trial had a significant effect on subjects’ accuracy (day: χ^2^_1_ = 6.040, *p* < 0.05, Fig. 2; trial: χ^2^_1_ = 13.322, *p* < 0.001): bearded dragons’ performances increased day after day and trial after trial (within the day). No interaction was statistically significant (all *p*-values > 0.201).

At the population level there was no left-right bias in the direction bearded dragons entered the cylinder (mean ± SD: 49.38 ± 20.43%; one-sample *t* test: *t*_7_ = −0.087, *p* = 0.934). However, individuals varied considerably in the degree of lateralization and Pearson correlation revealed a positive significant correlation between bearded dragons’ accuracy and lateralization (*r*_6_ = 0.898, *p* < 0.01) suggesting that more lateralized subjects had an advantage in solving the task and reaching the food.

### Hermann’s tortoise

With the opaque cylinder, male tortoises entered the cylinder in 98 ± 16% of trials (mean ± SD). Their mean time required to enter the cylinder was 201 ± 392 s (mean ± SD). Instead, female tortoises entered the cylinder in 98 ± 20% of trials (mean ± SD). Their mean time required to enter the cylinder was 302 ± 494 s (mean ± SD). We found no significant difference between the sexes in the time to enter the cylinder (*F*_1,150_ = 0.081, *p* = 0.777).

With the transparent cylinder, tortoises performed 28.13 ± 27.47% correct trials (mean ± SD; females: 34.17 ± 29.16%; males: 22.08 ± 24.49%) in which they retrieved food without touching the cylinder’s surface (correct trials). The mean time required to complete a trial regardless of outcome, was 106.61 ± 137.46 s (mean ± SD; females: 145 ± 184; males: 68 ± 35). These data seem to suggest that females have higher performances in terms of correct choices but take longer to complete the task and reach the food inside the cylinder.

**Fig. 3:**
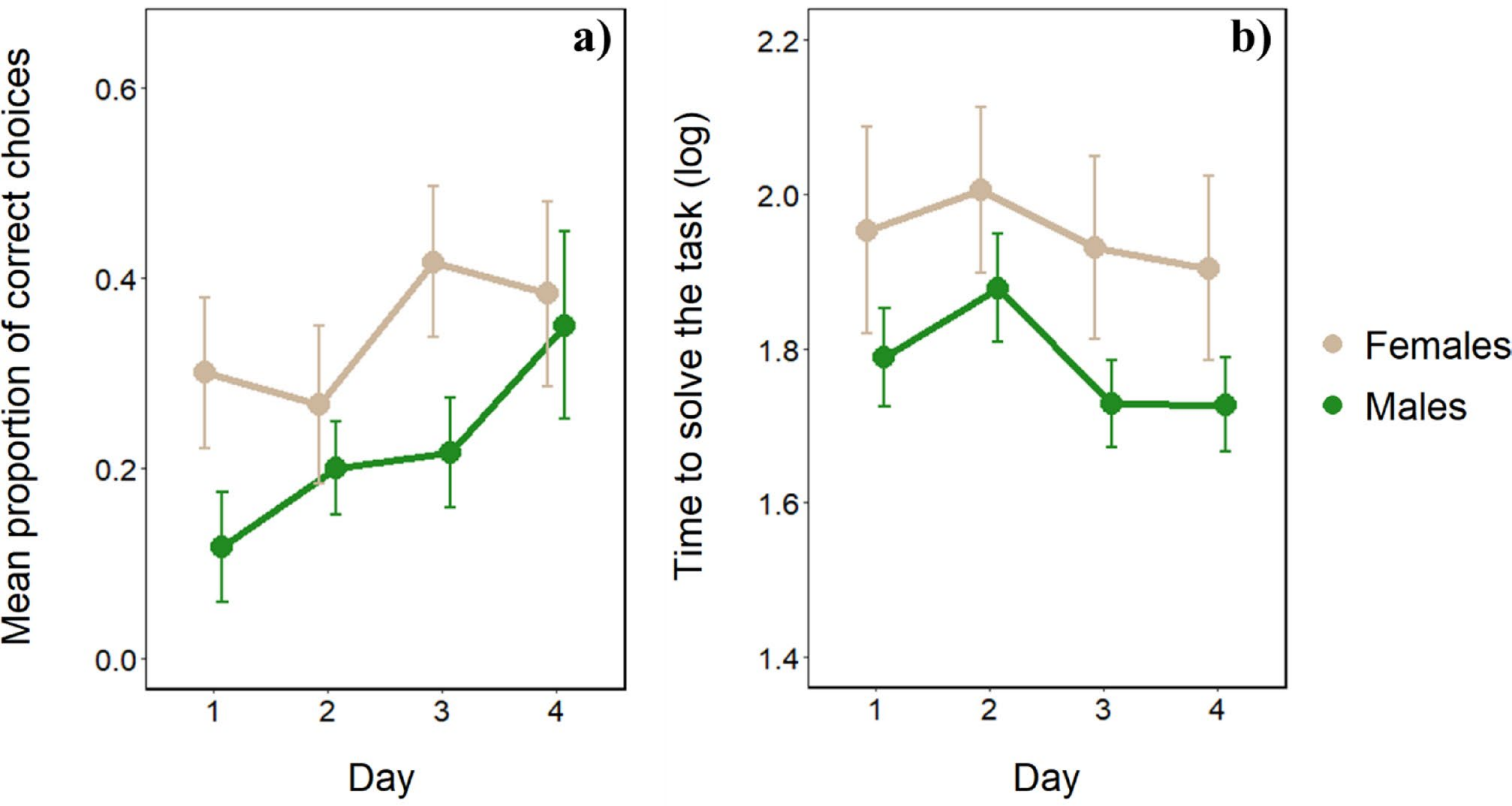
Comparison of the performances of females’ and males’ tortoises in the cylinder task. (**a**) Mean proportion of correct trials in which tortoises did not contact the cylinder (mean ± SE), and (**b**) time to solve the task (mean ± SE) over the four days of the test phase.

The GLMM revealed a significant effect of the time on subjects’ accuracy (χ^2^_1_ = 26.358, *p* < 0.001): the longer the time to reach the food, the lower the performance. Moreover, females’ performance was higher compared to males’ performance (χ^2^_1_ = 3.957, *p* < 0.05; Fig. 3). Also, day had a significant effect on subjects’ accuracy (χ^2^_1_ = 6.737, *p* < 0.01; Fig. 3) but not trial (χ^2^_1_ = 2.248, *p* = 0.134): tortoises’ performances increased day after day but not trial after trial (within the day). No interaction was statistically significant (all *p*-values > 0.101) with the exception of time × day (χ^2^_1_ = 9.808, *p* < 0.01): given the same amount of time needed to complete the task, day after day, tortoises’ performance was higher.

At the population level there was no left-right bias in the direction tortoises entered in the cylinder (males: mean ± SD = 42.50 ± 17.37%, one-sample *t* test: *t*_11_ = −1.494, *p* = 0.163; females: 45.00 ± 25.55%, *t*_11_ = −0.736, *p* = 0.477; all individuals: 43.75 ± 20.28%; *t*_23_ = −1.510, *p* = 0.145). A large variation in the degree of lateralization was observed also in tortoises and the Pearson correlation revealed a positive significant correlation between accuracy and lateralization (*r*_22_ = 0.405, *p* < 0.05) suggesting that, even for tortoises, more lateralized subjects had an advantage in solving the task and reaching the food.

### Interspecific comparison

When comparing tortoises’ and bearded dragons’ performance with the opaque cylinder, the LMM revealed that subjects took significantly longer to enter the cylinder in the first training day (*F*_1,281_ = 5.080, *p* < 0.05). Moreover, performances did not significantly change between the two species (*F*_1,30_ = 0.183, *p* = 0.186) but there was a significant difference between the curves of the two species (*F*_1,281_ = 0.037, *p* < 0.01) with tortoises taking significantly less time in the second day whereas bearded dragons did not decrease in the time to enter the opaque cylinder.

**Fig. 4:**
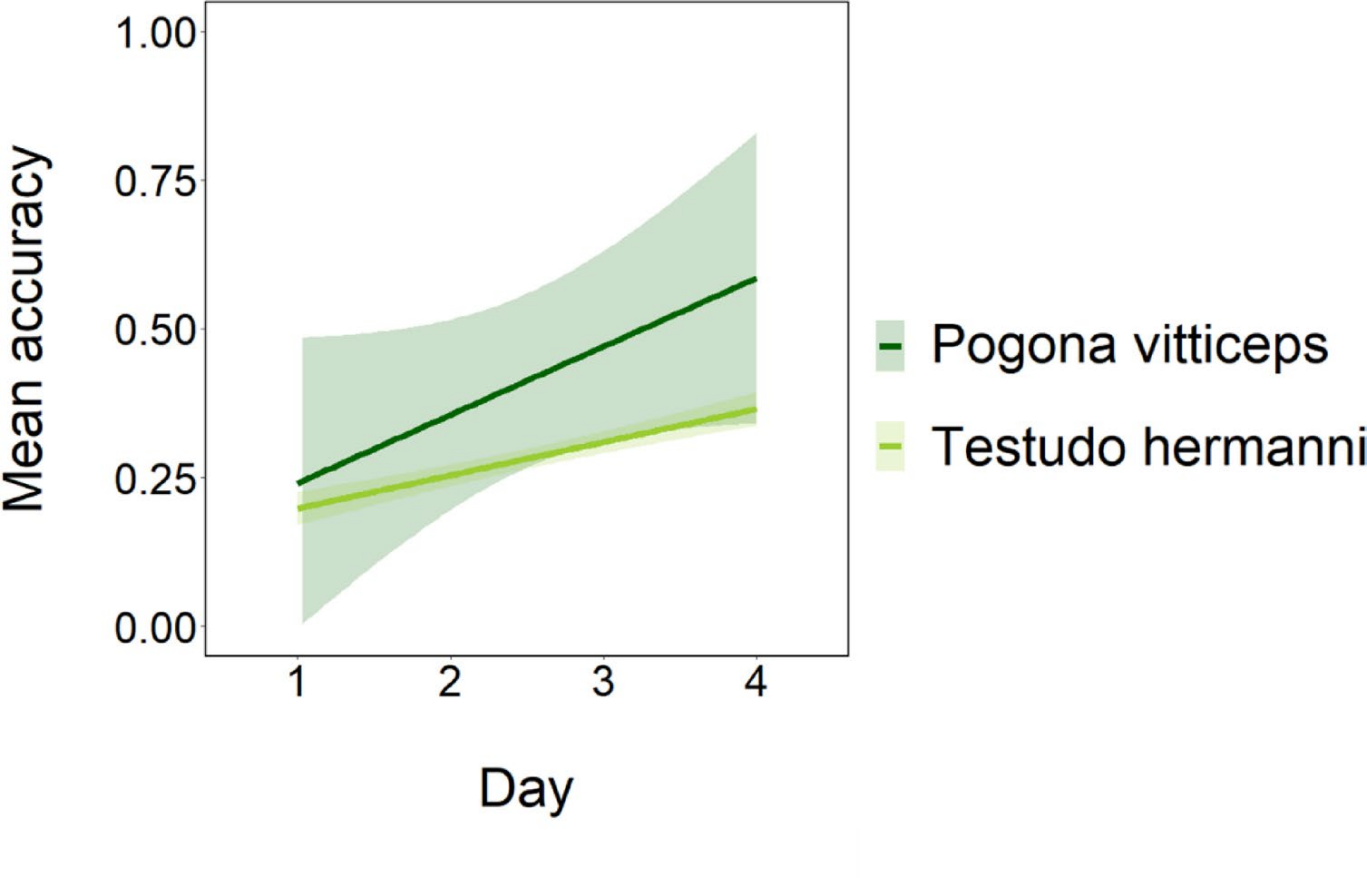
Comparison of mean accuracy between the two species. The shaded areas represent 95% confidence intervals around the estimated effects.

When comparing tortoises’ and bearded dragons’ accuracy with the transparent cylinder, the GLMM revealed that bearded dragons had a significant higher proportion of correct choices (χ^2^_1_ = 5.319, *p* < 0.05; Fig. 4). Moreover, performances significantly increased day after day (χ^2^_1_ = 19.843, *p* < 0.001; Fig. 4) with no significant difference between the performance trajectories of the two species (χ^2^_1_ = 0.808, *p* = 0.369; Fig. 4).

**Fig. 5:**
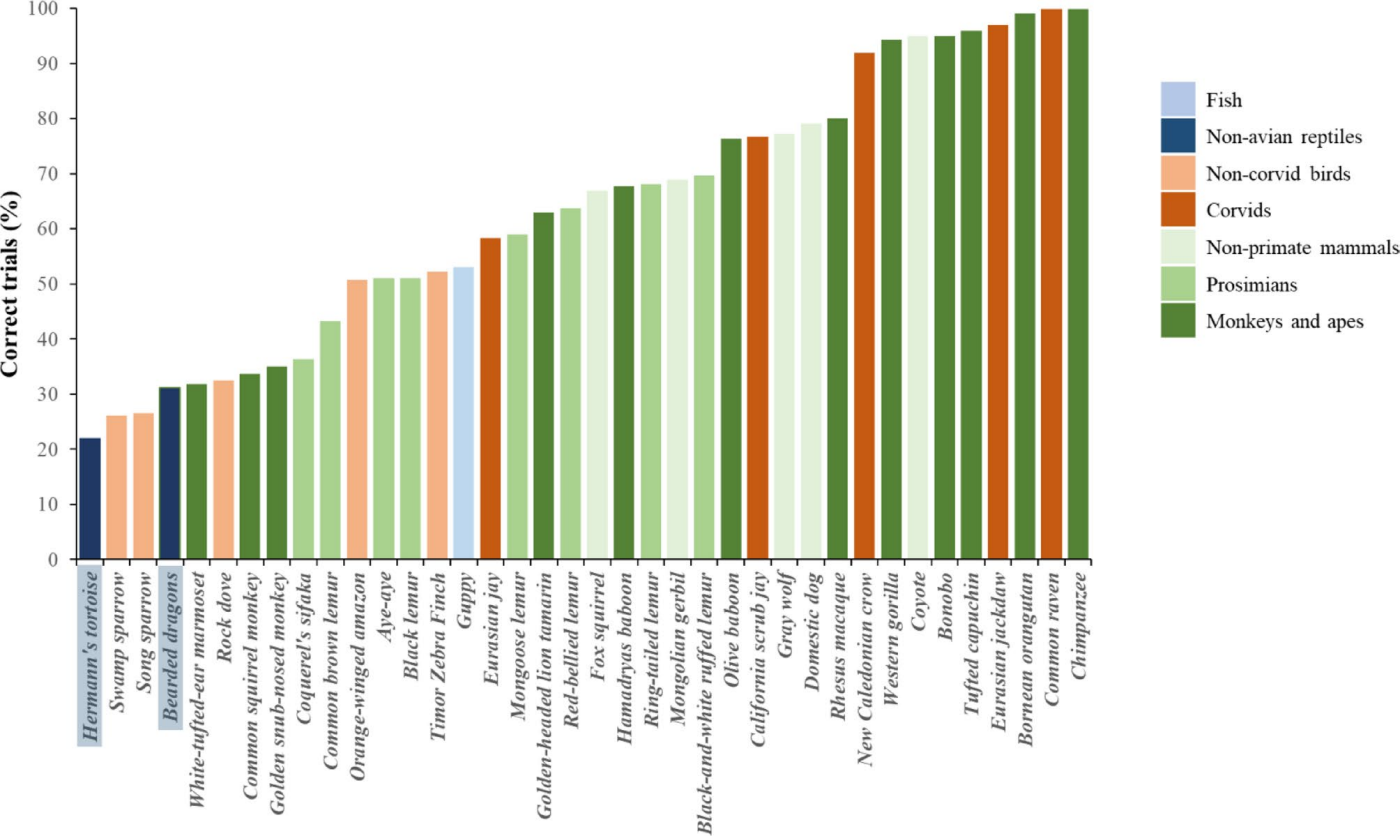
Comparison between the performance of Hermann’s tortoises and bearded dragons in the cylinder task (black bars) and that of different mammalian and avian species observed in the same task by MacLean et al.^9^ and Kabadayi et al.^10^, and fish observed by Lucon-Xiccato et al.^13^. Bars represent the mean percentage of correct trials. We used the performance of reptiles in the initial 10 trials to allow the comparison with the other species.

We also compared the performance of our subjects in the first 10 trials—an approach adopted in previous studies^9,13^—to allow direct comparisons with other vertebrate species observed in the same task. Bearded dragons achieved an average of 31 ± 28% correct trials, whereas tortoises reached 22 ± 19% correct trials in their first 10 trials. Figure 5 presents these results in the context of the performance of various mammalian and avian species reported by MacLean et al.^9^ and Kabadayi et al.^10^, as well as fish data from Lucon-Xiccato et al.^13^.

## Discussion

Our study provides the first data on inhibitory capacity in reptiles that can be used for direct comparisons with the other vertebrates studied so far. The procedure used in our study, the transparent cylinder test, is the most widely used for interspecific comparison of inhibitory control^9,10,36^.

Both tortoises and bearded dragons showed some capacity to inhibit a prepotent but ineffective response, and to temporarily move away from the goal to obtain the reward. As observed in the other vertebrates, performances increased day after day but in both reptiles, even after 4 days and 20 trials, most individuals performed less than 50% of trials correctly. Tortoises and bearded dragons showed a similar improvement rate in the course of the experiment but overall, bearded dragons made a significantly higher percentage of correct trials. This improvement over trials is consistent with findings in the vast majority of species observed with the transparent cylinder and similar tasks and raises the question of what underlies this temporal pattern. Since the training phase is intended to teach the appropriate motor response and minimize the role of learning during the test phase, it is important to consider what accounts for this temporal variation in performance. Van Horik et al.^8^ observed that pheasants with prior experience in a transparent barrier task performed better, suggesting that improvement across trials may reflect learning about the physical properties of the transparent material. However, Santacà et al.^7^ raised guppies under different conditions—either with or without exposure to detourable transparent obstacles—and found no effect of this manipulation on performance.

Another explanation, proposed by Brandão et al.^37^, is that early trials may be affected by neophobia or stress associated with the apparatus (e.g., the shift from opaque to transparent cylinder). As animals become more comfortable with the setup, their performance may improve simply because they are less distracted or hesitant. Lucon-Xiccato et al.^13^ further suggest that subjects might simply progressively improve the capacity to inhibit the prepotent response to reach directly through the barrier. Supporting this idea, several studies have shown that inhibitory control can be improved through repeated exposure or targeted training, even in contexts unrelated to transparent surfaces, such as computerized response inhibition tasks in children and adults^38,39^. Supporting this interpretation, tortoises—despite performing more poorly in the transparent cylinder—showed faster and more marked improvement than bearded dragons in the opaque cylinder phase, which represents a basic learning task.

Inhibitory capacities have been assessed across a wide range of vertebrate species using the transparent cylinder paradigm, revealing substantial interspecific variation both within and among vertebrate classes^9,10^. Species with large complex brains, such as apes and corvids, showed very high percentages of correct responses, in some cases approaching 100%^9,10^. In contrast, small passerine birds and micromammals showed much lower percentages, ranging between 25% and 40%^9^. As for teleost fish, the guppy (*Poecilia reticulata*) which has been extensively studied for inhibition capacities^8,13,40,41^, showed a performance of 50% in the transparent cylinder test, far below that of large-brained species but higher than almost all bird species and some mammals. There is some evidence that inhibition capacities may be similar or even greater in other teleosts^42,43^.

When we analysed the performance of the first 10 trials (the measure adopted in the other studies^9,14^, both reptiles performed poorly compared with other species. Bearded dragons achieved 31% correct responses, placing it at the lower end of the range observed across approximately 50 vertebrate species and well below the performance of teleost fish. Tortoises performed even worse, achieving only 22% correct trials. Inhibitory capacities have also been examined in scincid lizards using a task similar to the transparent cylinder test^21,22^. The five skink species tested showed an average performance of 34.6% correct responses, with interspecific variation ranging from 26 to 45%. These values were below those observed in teleosts and most endotherms. However, the experiments employed a different method, measuring detour ability around a perforated wire mesh barrier. The barrier’s characteristics are known to strongly influence performance in detour tasks^24–26^, but it is difficult to predict in which direction this may have affected skink performance. Studies that directly compared transparent barriers with other types of barriers—such as bars or grids—have found very different results, depending on the species observed^25,26,44^.

Currently, we can only speculate about the possible explanations for the difference between the two species of this study and about the reasons reptiles exhibit poorer inhibitory control abilities compared to warm-blooded vertebrates. The comprehensive comparative study conducted on over thirty species of endotherms suggests that absolute brain size is the primary predictor of these differences in inhibitory capacity^9^. In reptiles, both absolute and relative brain sizes are much smaller than in endotherms^11,45^. In this context, the lower performance of our two species compared to most mammalian and avian species aligns with the hypothesis of a positive relationship between inhibitory capacity and absolute brain size. Moreover, as neuron density increases with brain size across vertebrates, species with a higher encephalization index, such as mammals and birds, possess disproportionately larger numbers of neurons compared to species with smaller relative brain sizes, such as reptiles^11^. Therefore, the performance of the reptilian species examined so far could also support the hypothesis of a relationship between inhibitory capacity and neuron count. Additionally, other factors could be at play. For instance, the method of accessing food or other targets may influence inhibition; species using hands or paws, like primates, may exert better inhibitory control than those relying on direct access with the mouth, as manual manipulation allows for a more controlled approach^9^. Clearly, more data from additional reptilian species, especially larger reptiles, are needed before determining whether the hypotheses proposed for endotherms can be extended to all amniotes.

Understanding the exception represented by teleost fish may offer insights into the broader relationship between brain architecture and inhibitory control. These species have brains some orders of magnitude smaller than warm-blooded vertebrates, and significantly smaller than the reptiles studied^45^, yet they overperform various mammals, most birds, and all the reptiles examined so far in the cylinder test. A possible explanation for the exception represented by fish may lie in the unique evolutionary history of the bony fish genome and cognition. Over the last decade, it has been discovered that teleost fish possess several cognitive abilities once thought to be exclusive to land vertebrates. For example, they are capable of cooperating to achieve shared goals, acquiring new foraging and antipredator behaviours through social learning, using tools, engaging in sophisticated parental care, and even demonstrating behaviours that could be classified as cultural traditions (reviewed in^19,46,47^). It is now hypothesized that the evolution of such advanced cognitive abilities is linked to a whole-genome duplication event that occurred over 400 million years ago, after the teleost fish diverged from the lineage leading to terrestrial vertebrates. This duplication freed many genes to evolve new functions, which may explain both the remarkable biological diversity seen in modern fish and the development of complex cognitive abilities in small-brained organisms^20,48^. Other hypotheses have been proposed to explain this paradox. One possibility is that the general organization of their nervous system qualitatively differs from that of tetrapods, for example in glia/neuron ratios or neuronal density^49^. It was also suggested that fish may rival endotherms in certain cognitive abilities but lack entirely other functions - particularly domain-general processes – that are computationally demanding^50^. If confirmed, these findings suggest that teleost fish may represent a special case, shaped by unique evolutionary processes. Accounting for this exception may therefore help refine—not reject—the hypothesis that inhibitory control is broadly associated with brain size and neuron count, at least within amniotes.

Notably, the disparity observed between tortoises and bearded dragons is challenging to explain through brain size or neuron count hypotheses. If differences in brain size or neuron number exist, they would likely favour tortoises, suggesting that in this case other factors are involved^8,11^. For instance, an effective inhibitory control system may have evolved in some species but not in others due to ecological factors and the specific ways in which animals interact with their environment. For example, MacLean and collaborators^9^ found that, in addition to brain size, inhibitory control abilities positively correlate with diet breadth. They suggested that a more diverse and complex diet could drive the evolution of larger brains and advanced cognitive functions, which in turn may have facilitated the emergence of more sophisticated inhibitory capacities^9^. Alternatively, diet type may have directly influenced the evolution of more efficient inhibitory abilities, as species with broader diets face complex decision-making in diverse foraging contexts. This ecological factor could help explain the interspecific difference observed in our study, given that Hermann’s tortoise is almost exclusively herbivorous^51,52^, while the bearded dragon exhibits a more varied diet that ranges from primarily plant-based in some populations to others in which arthropods—and occasionally small vertebrates—contribute significantly to overall intake^53,54^.

Finally, both individual and species differences may be mediated by non-cognitive factors, such as feeding motivation and body condition^55^, or prior experience with transparent barriers^8^. Motivation has been shown to affect performance in many cognitive tasks^56,57^. In detour tests in particular, some species exhibit poorer performance when approach motivation is high, as individuals may find it harder to suppress the impulse to reach directly for the goal^40,55^. However, controlling feeding motivation in comparisons between phylogenetically distant species is extremely challenging, as they can differ significantly in physiology, energy requirements, diet, and ecology. Using the time taken to reach the food during the training phase as a proxy for feeding motivation, we found that tortoises were faster than bearded dragons, which is compatible with the feeding motivation hypothesis, although latency to reach the food can also be influenced by factors such as motor speed, exploratory tendency, or problem-solving strategies unrelated to motivational state.

As for prior experience with transparent barriers, its effect has been observed in some species but not in others^8,41,58^. Neither of the two species in our study had previous experience with detour tasks involving transparent obstacles. The bearded dragons were housed in vivariums which had a transparent barrier at the front, while tortoises were kept in large semi-transparent enclosures (e.g., wire mesh). However, we do not consider this difference to be critical, as individuals of both species had never experienced the possibility of passing beyond the walls of their enclosure. Instead, tortoises, which are known to encounter difficulties with transparent surfaces, tend to rely more on physical strength to navigate obstacles in their natural environment. For instance, research indicates that tortoises in dense vegetation environments primarily use force to push through obstacles^59^. Moreover, when confronted with physical barriers, such as steep steps, tortoises do not attempt sophisticated detour routes but rather persistently use their body strength to overcome these barriers^60^. This reliance on force over detour may also apply to artificial transparent barriers: tortoises may not interpret transparent obstacles as true barriers, resulting in behaviour focused on direct physical engagement rather than detouring or inhibiting prepotent responses.

Sex differences in cognitive performance have been reported in many vertebrates. These differences span a wide variety of cognitive functions, including numerical abilities, spatial cognition, problem-solving skills, etc^61–66^. With the exception of spatial abilities, where males generally outperform females, findings across other cognitive domains indicate that either sex may excel depending on the function and species studied. Recent studies have also reported sex differences in executive functions, a particular class of advanced cognitive processes, including cognitive flexibility, working memory, and inhibitory control, that enable the control of goal-directed behaviour^67–70^. Specifically, regarding inhibitory control, sex differences have been documented across a range of mammals, fish, and birds, sometimes favouring females and other times favouring males (reviewed in^63^). In this study, we found that sexual differences in executive functions exist also in reptiles. In the only species, Hermann’s tortoise, for which the sex ratio and sample size allowed for such an analysis, we found that females outperformed males in the cylinder test. The temporal pattern of performance suggests that this difference is not due to learning differences, as females showed superior performance from the beginning of the test, and both sexes improved at a similar rate over time.

Cognitive sex differences are generally thought to arise from differential selection pressures driven by ecological sex differences or by varying mating and reproductive roles. However, it is often challenging to pinpoint the exact cause of such differences in a species unless its life history, mating system, and any sex differences in foraging or other ecological aspects are known in detail. As far as we know from the biology of the Hermann’s tortoise, there are no significant trophic niche differences between the males and females, and the species’ mating system does not differ markedly from other vertebrates with a promiscuous mating system and no parental care^52,71,72^. Sex differences in cognitive tests may also stem from non-cognitive factors. Although the procedure was identical for both sexes, motivation could differ. For instance, field studies show that females invest more in reproduction due to the energy costs of egg production, resulting in poorer body condition during the breeding season, reduced annual survival, and shorter lifespan compared to males^73,74^. However, in captivity, where food is provided ad libitum and foraging can occur continuously, it is unclear whether this would lead to different feeding motivations. In any case, the direction of this effect would contradict the relationship between motivation and performance observed in other species^40^.

Other factors may also contribute to the observed difference. Sexual size dimorphism, a common feature in many reptile species, may also contribute to sex differences in inhibitory control by affecting brain size. In wild Hermann’s tortoises, males are generally smaller than females, though they possess proportionally larger heads. However, whether this reflects differences in absolute or relative brain volume remains to be determined^69^. In our study, we tried to match body size between the sexes as much as possible, and indeed, there were no significant differences in size between males and females.

Males and females may also differ in personality traits or cognitive styles^75^. Our data show that, on average, females took approximately twice as long as males to reach the food inside the cylinder, yet achieved higher performance in terms of correct trials. It remains unclear whether these two variables are causally linked. One possibility is that males and females adopt different approaches to the task, with females being more cautious and accurate due to slower responding. Alternatively, males may be more impulsive, which could result in both faster trial completion and a reduced ability to inhibit the prepotent response to reach directly through the transparent barrier.

An interesting finding of this study is the positive correlation, observed in both species, between an individual’s degree of lateralization and its performance in the cylinder test. The nature of this relationship remains unclear. Recent debates have focused on the evolutionary advantages of brain lateralization, which may confer adaptive benefits by enabling the simultaneous processing of different types of information in the two hemispheres^28,76,77^. This ability could help animals manage limited attention by performing two cognitive tasks at once, for instance feeding or mating while remaining vigilant for predators. In our case, it is difficult to argue that the experimental situation required sharing attention between two simultaneous tasks unless we assume that being in an unfamiliar environment outside of their home cage triggered automatic vigilance for potential predators. There are, nonetheless, several other cases like this where it seems challenging to invoke the advantage of more efficient multitasking to explain the superior performance of lateralized animals^28,78^ suggesting that other advantages must contribute to explain the ubiquity of lateralization. Another classic hypothesis for the ubiquity of lateralization is that it helps avoid the simultaneous activation of incompatible responses that might arise from symmetrical duplication of brain areas in both hemispheres. One situation where this might be relevant is when an animal needs to move around an obstacle to reach a goal. Since detouring to the left or right are functionally equivalent responses, a perfectly symmetrical nervous system could lead to simultaneous activation or mutual inhibition, potentially interfering with the execution of the response or at least slowing the decision-making process. In this sense, the influence of lateralization may not be on inhibitory control per se, but rather on the execution of the specific task in the transparent cylinder test. To test this hypothesis, one would need to assess inhibitory control using a task that does not involve spatial right-left components.

In conclusion, as with warm-blooded vertebrates, reptiles appear capable of inhibiting prepotent but inappropriate responses to a given context. By employing the same test used across a wide range of vertebrates, we were able to compare the inhibitory control of reptiles directly with that of other species. Our findings show that the inhibitory control abilities of reptiles, as measured by the transparent cylinder test, are lower than those of other amniotes observed so far. These results align with the prevailing hypothesis that the size and complexity of the nervous system are the primary predictors of inhibitory control abilities. However, the existence of significant exceptions to this rule among vertebrates suggests that species-specific ecological factors or non-cognitive variables may also play an important role in determining interspecific differences. This study confirms previous findings in other species, demonstrating that sex differences in inhibitory capacities also exist in non-avian reptiles and highlights for the first time that hemispheric lateralization can enhance cognitive functions, including inhibitory control, in cold-blooded amniotes.

## Electronic supplementary material

Below is the link to the electronic supplementary material.

Supplementary Material 1

## Data Availability

The authors confirm that the data supporting the findings of this study are available within the article’s supplementary materials.
